# New insight into RNA biomarkers in neuropathic pain: a clinician–neuroscientist roadmap to translational testing and treatment monitoring a clinical review

**DOI:** 10.3389/fpain.2026.1874336

**Published:** 2026-07-16

**Authors:** Amol Soin, Massab Khaira, Aviraj Soin, Dhilen Soin, Shreyas Shah, Sabrina Tolppi, Anubhav Tripathi

**Affiliations:** 1 The Ohio Pain Clinic, Centerville, OH, United States; 2 Lilac Biosciences, Providence, RI, United States; 3 Biomedical Engineering, Brown University, Providence, RI, United States

**Keywords:** chronic pain, genetic editing, genetic modification, molecular biomarker, neuropathic pain, neuropathy, precision medicine, RNA biomarker

## Abstract

Neuropathic pain affects an estimated 7%–10% of the global population and imposes an annual economic burden exceeding $600 billion in the United States alone. It lacks robust objective biomarkers; current diagnosis relies heavily on subjective reporting and heterogeneous phenotypes. Currently utilized pain assessment tools include the brief pain inventory (BPI), numerical rating scales (0–10 pain scores), and the visual analog score (VAS), which depend on patient-reported outcomes and are influenced by social, psychological and contextual factors. This subjectivity contributes to heterogeneous phenotyping and variability (>30%) towards the treatment response. Emerging transcriptomic and epitranscriptomic evidence suggests that RNA-based biomarkers may offer a biologically sound and objective approach to understanding and managing pain by capturing underlying molecular mechanisms. Therefore, the present clinical review focused on RNA biomarker classes (mRNA, miRNA, lncRNA, RNA editing, RNA modifications) and proposes a clinically deployable testing system for diagnosis, stratification, and treatment monitoring, since there are no FDA-approved RNA-based biomarkers for pain. Therefore, this review synthesizes evidence from immune-cell transcriptomic meta-analysis (TCL1A/ERAP2), dorsal root ganglion (DRG) and central nervous system gene expression patterns (EFNB2, GABBR1, NCAM1, SCN11A)/brain genetic architecture via single-cell omics integration, and atlas-driven frameworks, like iPain single-cell atlas of pain chronification and nociceptor senescence. Additional sources include studies on RNA editing mediator adenosine deaminase acting on RNA2 (ADAR2), clinical and translational evidence supporting miRNA biomarkers, and lncRNA axes (NEAT1/miR-183-5p; H19/miR-141) as tissue-specific regulatory nodes. Additionally, m6A epitranscriptomic modifications regulated by the METTL3/METTL14 writer complex and FTO/ALKBH5 erasers, with site-specific methylation of GRIN2B mRNA shown to upregulate GluN2B in dorsal horn neurons and augment central sensitization. These biomarkers also demonstrate potential utility as pharmacodynamic readouts in drug and neuro-modulation trials. Additionally, an emerging RNA workflow technology pathway leveraging rapid low-input RNA based assays was also explained. All evidence supports the idea that these biomarkers can provide complementary insight into the mechanisms underlying pain. Although current evidence supports the feasibility of RNA-based biomarkers as indicators of key biological processes, however, the current pain biology score remains at the theoretical model stage and has not been validated through *in vivo*, *in vitro* or clinical trials.

## 1 Introduction

### 1.1 The unmet need: objective measures in chronic pain

Neuropathic pain is a chronic pain condition, resulting from damage to the somatosensory nervous system, which significantly increasing the burden on patients, affecting approximately 6.9%–10% of the general population, and also increasing the burden on healthcare systems (1, 2). It can be described as stabbing, burning, electrical sensations, or tingling, occurring spontaneously or through allodynia or hyperalgesia (2). Most commonly, neuropathic pain occurs in trigeminal neuralgia, diabetic neuropathy, and post-therapeutic pain (3). Causes of central pain may be cerebrovascular injuries, trauma, multiple sclerosis, tumors, and Parkinson's disease (4). In addition, multi-complex disorders such as complex regional pain syndrome (CRPS) are rare chronic neurologic conditions that induce neuropathic pain, as they can alter the blood flow in lower limbs (5, 6). Despite significant advances in understanding its underlying mechanisms and pain medicine, challenges persist in achieving effective treatment due to its clinical manifestations (7).

A central challenge in the management of neuropathic pain is the lack of objective biological measures that can reliably quantify pain (8, 9). Usually, the neuropathic pain assessment begins with the patient's medical history, using self-reported pain scales to quantify pain intensity, conducting neurological assessments and measuring sensory thresholds (10). The assessment tools, like Leeds Assessment of Neuropathic Symptoms and Signs (LANSS), Douleur Neuropathique en 4 Questions (DN4), Neuropathic Pain Questionnaire (NPQ), pain DETECT, ID pain, Neuropathic Pain Scale (NPS), and Brief Pain Inventory (BPI) are effective for capturing subjective pain experience, however, these screening tools can wrongly suggest the pain condition and cannot replace the clinical examination (11). These tools can be helpful in distinguishing perceived pain into neuropathic and non-neuropathic pain, and therefore patients with mixed pain or with different underlying conditions can still pose a diagnostic challenge (12). This variability further complicates the stratification of patients and the evaluation of treatment efficacy. Critically, heterogeneous phenotyping driven by this subjectivity inflates placebo response rates to 30%–50% in neuropathic pain trials, directly contributing to a 90%+ Phase II-to-III attrition rate for analgesic candidates over the past two decades (11).

Conventional diagnostic modalities, like conventional nerve conduction studies, functional magnetic resonance, quantitative sensory testing (QST), and neuroimaging (13–15), provide additional insights, however, these modalities also have limitations. Structural imaging often shows poor correlation between the severity of pain symptoms and radiological findings on neuroimaging, particularly Magnetic Resonance Imaging (MRI) and ultrasound, and they should not be intended to define a precise diagnosis (16). Similarly, QST provides a systematic approach for the assessment of somatosensory function, however, limitations exist in its application, interpretation and broader implications. These limitations include variation in QST methodologies and their generalizability to pain modalities other than neuropathic pain and small fibre neuropathy (17). In addition, QST cannot localize the exact source of dysfunction alongside the neuroaxis (17). These challenges highlight a crucial gap between biological understanding and clinical observations. Therefore, the concept of biological measurements in pain has gained increasing attention.

**Figure 1:**
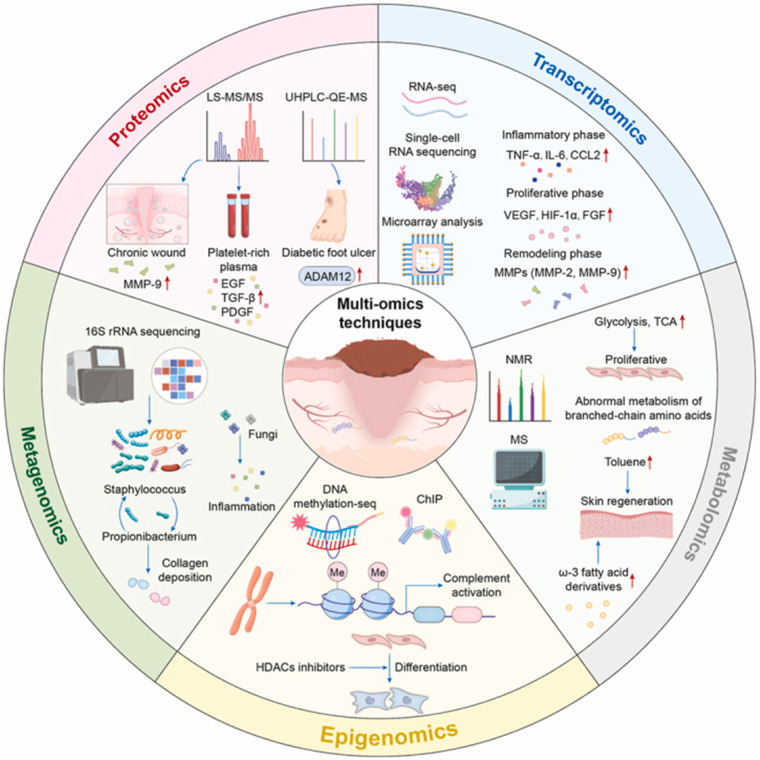
Application of multi-omics in the tissue repair and regeneration CC BY-NC-ND license (http://creativecommons.org/licenses/by-nc-nd/4.0/) (20).

Due to advancements in system biology and molecular technologies, which have successfully linked biology and mathematics, this approach is increasingly powerful in computational and molecular methods and plays a significant role in tackling the complex mechanisms underlying human disease (18). Among these approaches, multi-omics, which is represented by genomics, single-cell transcriptomics, transcriptomics, spatial transcriptomics, epitranscriptomics metabolomics, and proteomics, these approaches has a wide application in the study of human diseases and also provide very useful avenue for the study of underlying pathophysiology of chronic pain and has a significant role in the tissue repair and regeneration, as described in Figure 1 (19–21). These developments have opened new opportunities for identifying molecular signatures associated with specific pain states.

Among these emerging approaches, Ribonucleic Acid (RNA)-based biomarker, like non-coding RNAs (ncRNAs), including microRNAs (miRNAs), circulating RNAs (circRNAs), long non-coding RNAs (lncRNAs), and small interfering RNAs (siRNAs), have gained increased attention because they provide a dynamic snapshot of cellular response and gene activity (22). In addition, these ncRNAs modulate the expression of genes at transcriptional and post-transcriptional levels, which effectively altering pathways associated with pain (22). RNA expression profiles can also alter responses to injury, neural plasticity, and the inflammasome (22, 23). Meanwhile, with the aid of RNA sequencing, RNA profiling, which is increasingly scalable and clinically more feasible, can further improve the clinical understanding of the diagnosis and management of neuropathic pain (24). Moreover, RNA-based biomarkers occupy a uniquely translational position as RNA expression reflects real-time cellular responses to injury, inflammation, and therapeutic intervention with a temporal resolution (minutes to hours for mRNA induction; hours to days for epigenetic remodeling).

Critically, ultra-low-input RNA sequencing now enables transcriptome quantification from as few as 100–500 cells using advanced workflows such as single-tube direct lysis protocols or specialized low-input RNA sequencing methods (25, 26). This lowers the practical threshold for liquid biopsy application, shifting the requirements from whole tissue down to peripheral blood mononuclear cells (PBMCs) and plasma-derived exosomes (23, 24, 27). However, these ultra-low applications are primarily restricted to specialized research settings. Whereas routine bulk RNA sequencing methods typically require a detection baseline of 1,000 cells to ensure sufficient yield post-extraction and robust reproducibility (28, 29).

### 1.2 Why RNA? A unifying layer between genotype, cell state, and phenotype

RNA plays a central role in molecular biology, as it not only serves as a messenger between proteins and DNA but also has a significant role in performing diverse regulatory functions (30). In case of neuropathic pain, RNAs that are dysregulated following injury could contribute to nociceptive plasticity and the generation of neuropathic pain (31). Changes in expression of RNA are therefore actively involved in the neuropathic pain regulation by affecting the processing and transmission of signals associated with neuropathic pain (32).

Numerous classes of RNA molecules play active roles in the biology of pain. For instance, mRNA represents the most direct readout of the programs associated with gene expression in cells, and disturbances in mRNAs diminish pain-associated behaviors (33). Transcriptomic studies in peripheral nerves, spinal cord, and dorsal root ganglia, have identified widespread alterations in expression of mRNA following nerve injury (34, 35). Meanwhile, animal studies demonstrated that signaling pathways upstream of mRNA translation, like ERK and mTORC1, are upregulated, and their inhibition effectively alleviates pain (35). The most substantially enriched biological processes among the upregulated mRNAs were also involved in apoptosis, immune system processes, inflammation response, defense response, and sensory perception of pain (36). In addition to mRNA, non-coding RNA molecules have emerged as key regulators of gene expression mechanisms in the nervous system (37). Among non-coding RNAs, miRNAs have a fundamental role as post-transcriptional regulators (38). Several animal studies have demonstrated that numerous miRNAs play a significant role in the development of neuropathic pain at various stages of the nociceptive pathways, including synaptic transmission, neuronal excitability, communication with non-neuronal cells, and intracellular signaling (39). Alterations in the miRNAs, including miR-101, miR-132, and miR-199a, have a clear association with neuropathic pain (40). Also, alteration in miRNA expression patterns may impact the progression of inflammatory and neuropathic pain by controlling neuroinflammation, nerve regeneration, and influencing the expression of normal ion channels (41). Similarly, long non-coding RNAs (lncRNAs) are another important class of regulatory transcripts that play a critical role in several biological functions (42). These molecules can influence the expression of genes through different mechanisms, such as organization of chromatin, post-transcriptional control, and transcriptional regulation (42). Emerging evidence suggests that lncRNAs participate in responses to neuronal injury and neuroinflammatory signaling, potentially shaping the transcriptional landscape of circuits associated with pain (43). Additionally, aberrant expression of circRNA homeodomain-interacting protein kinase 3 (circHIPK3) within dorsal root ganglion (DRG) and serum, alongside dysregulation of lncRNAs such as nuclear enriched abundant transcript 1 (NEAT1), X-inactive specific transcript (XIST), and small nucleolar host gene 1 (SNHG1) has been significantly impact the pathogenesis of pain development. Furthermore, alteration of specific circular transcripts such as ciRS-7, zinc finger protein 609 (circZNF609), circ_0005057, and circAnks1a in spinal cord actively contributing to nociceptive signaling pathways (44). Furthermore, alteration of ncRNAs expression is considered as potential biomarkers and therapeutic targets for managing neuropathic pain (45). The RNA N6-methyladenosine (m6A) and inosine are being explored as key targets for clinical biomarkers. It is a critical mRNA modification that regulates its localization and stability in dorsal root ganglion neurons in animal model, and also observed an increased m6A abundance, along with upregulated expression of methyltransferases and decreased expression of demethylases, which can be associated with neuropathic pain (46).

### 1.3 Scope and audience

This review aims to provide valuable insights for translating RNA biomarker discoveries into practical tools for the diagnosis and treatment monitoring of neuropathic pain. This review is intended for a multidisciplinary audience, which includes molecular biologists, clinicians, and neuroscientists. By integrating basic neuroscience and clinical medicine, this review seeks to bridge the gap between real-world clinical applications and laboratory discoveries. In addition, this review will examine emerging evidence for RNA-based biomarkers in different compartments, including cerebrospinal fluid, peripheral blood, and neural tissues. It will further explore advances in transcriptomics, computational biology, and single-cell technologies, which can accelerate the discovery and validation of biomarkers.

## 2 Biological rationale: chronic pain as a state of persistent cell/circuit reprogramming

Pain is an active, multidimensional pathophysiological process, not merely a consequence of prolonged nociception but a state of persistent neural and cellular circuit reprogramming (47, 48). Following injury to tissues or nerves, glial cells, including satellite cells or astrocytes, undergo alterations in their phenotypes, thereby amplifying painful stimuli, which is mediated by chemokines, cytokines or ATP signaling (49). In addition, these glial cells lead to central and peripheral sensitization via enhanced excitability and dysregulation of ion channels. In parallel, epigenetic mechanisms, like histone modifications and DNA methylation, alter gene expression (BDNF, SCN9A) associated with pain, establishing a long-term transcriptional predisposition to chronic pain (47).

### 2.1 Peripheral drivers: DRG nociceptor subtypes, state transitions, and chronification programs

The dorsal root ganglia (DRGs) and trigeminal ganglia (TG) are the first target sites, which contain the cell bodies of primary sensory neurons, including nociceptive neurons. After painful injury, DRG cell bodies may undergo maladaptive molecular changes due to these primary sensory neurons (50, 51). These changes result in hyperexcitability and hypersensitivity of sensory neurons and are critical for the onset and maintenance of chronic pain (50). Unlike neurons in the central nervous system, DRG and TG lie outside the blood-brain barrier, making them more accessible targets for molecular and pharmacological interventions (51).

Recently, single-cell sequencing studies have identified nociceptor subtypes within the DRG, each with distinct functional roles in nerve regeneration and pain, with different molecular signatures (52). Mainly, these include peptidergic nociceptors, which express neuropeptides, like calcitonin gene-related peptide (CGRP), implicated in the neurogenic inflammation development, and are upregulated in the neuropathic pain condition (53). Non-peptidergic nociceptors associated with mechanical pain and myelinated A and C-fiber nociceptors, and evoke painful percepts, when activated selectively (54).

Following tissue injury, nociceptors undergo extensive functional and structural modifications, and such changes are essential for the development and maintenance of chronic pain (27). Subsequently, molecular adaptations also occur, including functional and expression changes in the ion channels, intracellular and receptors signaling pathways, as well as shifts in gene transcription, which modulate the processing of nociceptive signals (27). With the passage of time, this can lead to sensitization in which nociceptors exhibit lowered thresholds, increased spontaneous activity, reduced inhibition, and also manifestation with a remarkable plasticity of the somatosensory nervous system in response to neural injury (55). These changes represent the early stage of chronification programs, in which persistent gene expression patterns stabilize a hyperexcitable state (56). Such a program is an active and multidimensional pathophysiological process, not merely a consequence of prolonged nociception (47). Furthermore, these programs also involved in the epigenetic modifications in the interactions with satellite glial cells surrounding DRG neurons and altered receptor signaling (47). Together, these mechanisms are helpful in transforming transient nociceptive responses into pathological pain signaling.

### 2.2 Central mechanisms: brain circuit cell-type context of genetic risk

Peripheral sensitization initiates chronic pain, and long-term maintenance often relies on adaptations of the central nervous system. CRPS is a prototypical bridge between neuropathic pain and nociplastic pain, which is characterized by peripheral sensitization and neurogenic inflammation (5). Genetic and epigenetic factors play a significant role in the modulation of neuro-inflammation, which is involved in central and peripheral sensitization (57). Meanwhile, the perception of pain is ultimately shaped by distributed brain circuits that integrate sensory input with cognitive-evaluative, affective-motivational components, and contextual information (58). This integration further elucidates connectivity between cortical and subcortical structures to specific brainstem regions and provides a framework for the integration of nociceptive inputs with influences, which originates from top to down, which resulted in the appropriate modulation of these inputs to ensure the resultant experience of pain is appropriate for those specific circumstances (59).

Recently, studies combining GWAS with single-cell transcriptomic data have provided insights into the cellular context of chronic pain genetic risks (60). Pain-associated variants were found to be enriched in glutamatergic neurons, particularly in the hippocampal CA1–3, prefrontal cortex, and amygdala, while the human PEP transient receptor potential vanilloid 1 (hPEP.TRPV1/A1.2) neuronal subtype has demonstrated robust enrichment in humans (60). These nociceptors are associated with thermal, and inflammatory pathways related to pain (61). These analyses demonstrated that the risk of chronic pain converges on the brain's network of excitatory neurons and peripheral sensory neurons (62). Such outcomes suggest that inherited susceptibility may influence how these neurons respond to injury or sustained nociceptive input, potentially shaping long-term remodeling of the circuit. Furthermore, numerous genes, such as ephrin-B2 (EFNB2), neural cell adhesion molecule 1 (NCAM1), GABA-B receptor subunit 1 (GABBR1), and sodium voltage-gated channel alpha subunit 11 (SCN11A) have been in particular implicated in cervical DRG responses during transitions from acute to chronic pain (60).

## 3 RNA biomarker classes and what each adds clinically

Due to the molecular complexity of chronic pain, there is no single biomarker which is considered sufficient to capture the diverse biological processes involved in the initiation of pain, its chronification and treatment response (63). Rather, clinically, it is useful to use a composite of biomarkers comprising numerous measurements (63). Biomarkers, which are based on RNA provides a promising solution as they reflect dynamic cellular stress, activation of molecular pathways, and gene regulation (64). Different classes of RNA, including mRNA, miRNA, lncRNA, epitranscriptomic modifications, and RNA editing events, offer valuable biological insights associated with pain (22, 64). Together, these layers form a multilayer biomarker stack, where integration of these RNA-based biomarkers can improve diagnostic accuracy, enable mechanistic understanding and support precision medicine in research related to pain (Table 1).

**Table 1:** RNA biomarker modalities for chronic pain testing.

RNA class	Sample type	Clinical role	Outcomes (upregulation or downregulation)	miRNA diagnostic method and RT-qPCR validation approaches/Profiling technique	Refs.
miRNA	Serum	Diagnosis	miR-382-5p ↑	RT-PCR	(173)
miRNA	Biopsy	Expression	miR-124a and miR-155 ↑ and SIRT1 ↓	RT-qPCR	(174)
miRNA	Biopsy (tissue)	Stratification	miR-23a ↓	RT-qPCR	(175)
miRNA	Serum	Expression	miR-101 ↓	Taqman-RT-PCR	(176)
miRNA	Whole blood	Expression	miR-128 ↑, miR-155 and 499 ↓	RT-PCR	(177)
circRNA	Ipsilateral spinal dorsal horns	Expression	circ-363, 003724, 008008, 008973, 013779, 008646, 35215 ↑, while circ-106, 011111, 007419, 007512, 010913 ↓	Microarray, PCR	(178)
circRNA, mRNA	Postherpetic neuralgia skin	Expression	circRNA-66 ↑, circ68 ↓, and mRNA ↑	Microarray	(179)
lncRNA, mRNA	Spinal dorsal horn	Expression	745 mRNA and 139 lncRNA ↑	Microarray	(180)
lncRNA, miRNA	Whole blood	Expression	lncRNA NEAT 1 ↑, miR-183-5p, 433-3p ↓	RT-qPCR	(181)
lncRNA and miRNA	Serum	Expression	lncR-H19 and miR-141 ↓	RT-qPCR	(182)

### 3.1 mRNA signatures: state markers, pathway activation, and cell-type deconvolution

The expression of mRNA provides a direct readout of cellular states and active biological pathways, indicating its potential as a valuable indicator in the mechanisms involved in the disease (65). In neuropathic pain, transcriptional changes often occur in immune cells, DRG neurons, and the brain circuit involved in the nociceptive processing (66). Thus, the expression of gene signatures can be helpful in revealing how central and peripheral mechanisms contribute to the development of pain.

Genes like EFNB2, GABBR1, NCAM1, and SCN11A are actively involved in the initiation of pain, and these genes are particularly found upregulated (60). In addition, endoplasmic reticulum aminopeptidase 2 (ERAP2) has been shown to correlate with the severity of the pain (67). Meanwhile, EFNB2 is involved in axonal guidance and synaptic plasticity, suggesting its role in neuronal remodeling after injury (68). GABBR1, which is a subunit of GABA B receptors, which acts slowly and maintains the versatile regulators and inhibitory neurotransmission in the neuropsychiatric disorders and complex nervous behaviors (69). NCAM1 plays a significant role in numerous brain-associated biological processes, including axonal branching, neuronal migration, synaptogenesis, and fasciculation, and plays a critical role in synaptic plasticity (70). Meanwhile, the SCN11A gene encodes the sodium channel NaV1.9, which is functionally expressed exclusively in small-diameter peripheral nociceptors of the DRG and trigeminal ganglia. This channel is primarily involved in mediating persistent inflammatory pain after nerve injury or inflammation and serves as a baseline regulator of nociceptive threshold for pain signals (71). To date, only gain-of-function variants have been described for SCN11A (NaV1.9), in contrast to SCN9A (NaV1.7), which has both loss-of-function and gain-of-function variants and is often considered a gold standard for genetic validation.

Furthermore, a critical advantage of mRNA profiling is that it provides circulating immune transcriptomic data from peripheral blood, suggesting its role in mitochondrial dysfunction and neuro-inflammation (72). GWAS meta-analysis was performed for the identification of cell types and pathways relevant to the trait associated with chronic pain, and 461 clusters and 3,313 sub-clusters were identified and organized according to hindbrain, midbrain, and forebrain (73). Furthermore, cell-type deconvolution can be performed via computational approaches, which allowing researchers to infer the relative contributions of immune cell populations, inflammatory pathways, and neuronal signals, thereby capturing both whole-system and cell-centered level context (74). It is important to note that rigorous mRNA biomarker implementation requires standardized pre-analytical and analytical protocols. RNA Integrity Number (RIN ≥ 7.0) is a mandatory pre-analytical quality threshold; samples with RIN < 7 show 3′-biased degradation artifacts that distort differential expression estimates, particularly for long transcripts (>3 kb). Rigorous analytical validation extends beyond standard RIN metrics. To reliably quantify low-abundance transcripts and subtle epitranscriptomic marks in clinical settings, strict sequencing thresholds must be mandated. For whole transcriptome profiling, a minimum depth of 25–30 million paired-end reads should be required to minimize dropout of rare regulatory lncRNAs. For m6A-seq or ADAR editing analyses, depth must exceed 50 million reads to ensure sufficient coverage at targeted loci.

### 3.2 miRNA signatures: circulating biomarkers + treatment-responsive markers

miRNAs, small regulatory RNAs, are widely distributed, and target both the degradation of mRNA and suppression of protein translation by sequence complementarity between the miRNA and its target mRNA (75). Most miRNAs are transcribed from the sequence of DNA into primary miRNAs and processed into precursor miRNAs, and then maturation of miRNAs occurs. Meanwhile, the targeted mRNAs 3 untranslated regions (3 UTRs) are used for interaction with miRNAs, which actually induce mRNA degradation and translational repression (76). Because a single miRNA can regulate multiple genes simultaneously, miRNA signatures often capture coordinated regulatory networks associated with prognosis of the disease (77). These regulatory networks can play a significant role in understanding disease patterns, allowing researchers to modulate the expression of miRNA in response to usual therapy and also helpful in developing novel therapies (78).

In pain research, alterations in the circulating miRNAs in serum have gained considerable attention as a non-invasive clinical biomarker (79). Meanwhile, circulating miRNAs in small vesicles and exosomes mediate intercellular communication, which can be helpful for targeted interventions and the discovery of biomarkers (79). In addition, specific miRNA panels can be used to develop personalized patient profiles, which can be helpful in designing more specific therapeutic interventions (80). Notably, circulating miRNAs are also helpful for differentiating between nociplastic and nociceptive pain mechanisms, as well as for monitoring changes in expression levels in response to therapeutic interventions, providing opportunities for the monitoring of treatment (81). For instance, in patients, circulating miRNA alterations can be reversed following anti-inflammatory treatment with celecoxib, miRNA as possible mediators (82).

Meanwhile, evidence from fibromyalgia, a chronic musculoskeletal pain syndrome, further supports the clinical feasibility of miRNA biomarkers (83). Specific miRNA panels, such as miR-217 and miR-532 derived from peripheral blood mononuclear cells (PBMCs), which have been identified and subsequently validated using RT-qRCR, and these can serve as potential diagnostic biomarkers (83, 84). The validation demonstrates that miRNA biomarkers can be translated into standardized assays suitable for clinical laboratories.

Additionally, increasing evidence indicates that miRNAs have a significant role in numerous regulatory roles in the pharmacology of opioids and are actively involved in the opioid receptor regulation (85). miRNA also targets TGF-beta, angiogenesis, hypoxia, coagulation, inflammatory and immune system pathways (86). Moreover, miRNAs have also been proposed to regulate the expression of genes and degradation at the post-transcriptional level, including µ-opioid receptors as well as neuroplasticity and synaptic plasticity, in both the central and peripheral nervous system (87). Therefore, such biomarkers could help in identifying patients at risk of adverse responses to opioid therapy and guide safer strategies for pain management.

### 3.3 lncRNA signatures: mechanistic depth and stability

lncRNAs are widely expressed and play a key role in regulation of gene expression and can regulate the function and assembly of membrane-less nuclear bodies, modulate chromatin function, alter the translation and stability of cytoplasmic mRNAs and also interfere with signaling pathways (88). In addition, lncRNAs regulate transcription, stability of mRNA, splicing, mRNA availability, and post-translational modifications (89). Compared with other classes of RNA, lncRNAs often display high cell and tissue specificity, making them valuable for the identification of the molecular identity of specific immune cells or neuronal states (90). As more lncRNAs are being assigned to biological functions, the specificity of lncRNA expression is also increasingly recognized as a potential biomarker and for developing highly targeted therapeutic interventions (91).

In the context of chronic pain, lncRNA levels are found to be dysregulated in various chronic pain models, including neuropathic pain, and this dysregulation is responsible for the activation of various mechanisms, such as activation of miRNA, inflammatory cytokines, and these mechanisms play a critical role in the development of chronic pain (92). In addition, lncRNAs modulate the expression of genes at both transcriptional and post-transcriptional levels, affecting pathways, such as neuronal plasticity, inflammation, and sensory processing associated with pain (22). Furthermore, lncRNAs provide insights into brain region or DRG-specific regulatory mechanisms underlying pain chronification (93).

Recent evidence suggests that the axis of lncRNA alongside mRNA and miRNA plays a crucial role in the pathology and physiology of many diseases, including neuropathic pain (94). The potential mechanisms of this axis can be as follows: miRNA is sponged by lncRNA, which acts as a competitive endogenous RNA, and another involvement of lncRNA is keeping miRNA away from mRNAs, resulting in a reduction in the number of available miRNAs and helping to improve the translation of target mRNAs (94). Another possible mechanism is the degradation of lncRNA by miRNA, which affects diverse cellular processes, for instance, in human embryonic stem cells, lncRNA-RoR targets miR-145-5p, while the increasing concentration of miR-145-5p reduces lncRNA-RoR activity (95). In addition, lncRNAs can directly bind to complementary mRNAs at the mRNA-miRNA binding site, leading to the removal of the regulation of miRNA on mRNA (94). Such bridging may successfully capture both regulatory network dynamics and gene expression changes, improving diagnostic accuracy and biological interpretability.

### 3.4 RNA editing as a neuropathic pain mechanism and biomarker layer (ADAR2)

RNA editing represents an important step used to regulate the expression of genes via post-transcriptional mechanisms (96). More specifically, it involves adenosine (A) to inosine (I) RNA editing, which is catalyzed by ADARs (adenosine deaminases acting on RNA) on stranded RNA (97). Beyond ADAR-mediated editing, the broader epitranscriptomic landscape particularly N6-methyladenosine (m6A) modification exerts profound, dynamic control over pain chronification. The m6A stoichiometric balance is governed by a tripartite system of “writers” (methyltransferase complexes including METTL3 and METTL14), “erasers” (demethylases such as FTO and ALKBH5), and “readers” (such as the YTHDF protein family) which collectively dictate mRNA stability, splicing, and translational efficiency. Following nerve injury, the expression levels of these regulatory enzymes are significantly disrupted in the dorsal root ganglion (DRG) and spinal cord. For example, injury-induced downregulation of FTO or targeted upregulation of METTL3 can result in the hypermethylation of key nociceptive transcripts, amplifying their translational output independent of baseline mRNA abundance. Because these modifications can be precisely quantified using tools like LC-MS/MS to measure global m6A/A ratios or m6A-MeRIP-seq for transcript-specific locus mapping profiling the writer/eraser expression ratios offers a highly quantitative, post-transcriptional biomarker signature for neuropathic states. In neuropathic pain models, the patterns of RNA editing change following nerve injury, reflecting altered synaptic function and neuronal signaling.

Experimental studies have found that increased in expression of ADAR2 and decreased in expression of ADAR3 were observed in mice with injured DRG neurons, whereas in non-injured mice, nothing changed (98). Most notable editing targets include transcript encoding of the Glutamate α-amino-3-hydroxy-5-methyle-4-isoxazole propionic acid receptors (AMPARs) subunit GluA2, 5-hydroxytryptamine 2C receptor (5-HT2CR), and coatomer protein complex subunit α [COPA] (98, 99).

Genetic studies provide strong evidence for the functional relevance of these editing changes and for the disruption of mechanisms involved in RNA editing in tactile allodynia, which is a hallmark of neuropathic pain (98). Furthermore, the administration of fluoxetine, an antidepressant, can inhibit neuropathic allodynia after injury and in injured DRG, it successfully reduced the COPA site editing (98). These findings suggest that RNA editing signals may serve as treatment-responsive biomarkers, reflecting disease mechanisms and therapeutic effects.

### 3.5 RNA modifications/epitranscriptomics: “RNA state beyond expression”

Beyond RNA abundance and editing, RNA molecules undergo a wide range of chemical modifications collectively referred to as epitranscriptomic marks (100). RNA can undergo over 170 chemical modifications, including N6-methyladenosine (m6A) and 5-methylcytosine (m5C), and these modifications are also helpful in the regulation of RNA stability, cellular localization, and translation efficiency (101). In the context of A-to-I editing, this distinction between the regulatory machinery and the actual modification is critical for biomarker development. Relying solely on the expression levels of ADAR enzymes provides an incomplete, and potentially misleading, picture of the epitranscriptomic landscape. While ADAR mRNA or protein abundance indicates the potential for editing, it does not account for enzyme activation kinetics, dynamic subcellular localization, or substrate accessibility. Consequently, directly quantifying inosine levels and mapping their precise loci across the transcriptome yields a significantly more accurate functional readout. By employing advanced bioinformatics to calculate exact A-to-I editing frequencies at specific neuropathic target sites, clinicians can capture the true, real-time post-transcriptional state. This direct mapping of inosine coordinates provides a high-fidelity, highly specific quantitative biomarker signature that fundamentally outperforms inferences drawn from ADAR expression levels alone. In cases of neuropathic pain, epitranscriptomic changes can reflect inflammatory signaling, cellular stress, and metabolic alterations, as chronic pain involves complex interactions among immune responses, neuronal activity, and inflammatory pathways (102). Specifically, N6-methyladenosine (m6A) and inosine have emerged as highly quantifiable drivers of nociceptive plasticity in recent literature. For instance, m6A, the most abundant internal mRNA modification, is dynamically regulated in the dorsal root ganglion (DRG) and spinal cord following peripheral nerve injury. Studies demonstrate that dysregulation of m6A “writers” (e.g., METTL3) or “erasers” (e.g., FTO) leads to aberrant hypermethylation of specific pain-associated transcripts, such as voltage-gated sodium channels and inflammatory cytokines. This methylation fundamentally alters transcript stability and translational efficiency, driving neuronal hyperexcitability even when total mRNA abundance remains unchanged. Similarly, the generation of inosine through ADAR-mediated A-to-I editing plays a direct, stoichiometric role in central sensitization. A canonical example is the editing of the GluA2 subunit of the AMPA receptor at the Q/R site. In a healthy state, this site undergoes nearly 100% editing to tightly restrict calcium permeability. However, in chronic neuropathic pain models, a quantifiable reduction in this site-specific editing frequency directly increases neuronal calcium influx, driving persistent synaptic potentiation. By explicitly measuring these modifications, such as calculating absolute m6A/A ratios or quantifying the exact percentage of inosine conversion at the GluA2 Q/R locus, clinicians can access a highly specific, mechanistically grounded biomarker layer that directly reflects the chronification process. Therefore, RNA modifications may capture molecular states that are not detectable via gene expression analysis alone.

From a diagnostic perspective, epitranscriptomic profiling represents a next-generation biomarker strategy, extending beyond traditional transcriptomics and has the potential for early disease detection (103). In pain research, such modifications could provide insights into inflammatory signaling dynamics, neuronal stress responses, and treatment effects, thereby complementing expression-based RNA biomarkers (104). Furthermore, m6A quantification at pain-relevant transcripts detectable via low input m6A detection methods provides a next-generation biomarker layer capturing RNA state changes invisible to expression-level analysis alone.

## 4 Evidence base: what human studies already suggest is feasible

The growing interest in RNA-based biomarkers for pain is well supported by human studies demonstrating that molecular signatures of pain states can be detected in accessible tissues, like blood, and are linked to neural mechanisms of nociception (105, 106). Advances in single-cell sequencing, transcriptomics, and integrative genomic analysis have enabled scientists to identify consistent patterns of the expression of genes that are associated with pain subtypes, severity, and patient-specific biological characteristics. Notably, biomarker discovery is increasingly feasible in human populations.

### 4.1 Blood-based transcriptomics in chronic pain: conserved + sex-specific signatures

Blood-based transcriptomic profiling has increasingly attracted attention as one of the most promising strategies for identifying non-invasive biomarkers of chronic pain (107). Because blood is the main pipeline of the immune system and contains circulating immune cells that respond to neural injury signals and systemic inflammation, transcriptomic changes in these cells reflect underlying mechanisms responsible for the disease (108). Several large-scale cohort studies have analyzed peripheral blood gene expression profiles across different pain conditions, including fibromyalgia and neuropathic pain (107, 109).

A key finding from transcriptomic meta-analysis in the case of neuropathic pain condition is the presence of both sex-specific and conserved gene expression signatures (110, 111). Conserved signatures represent changes in the expression of genes that are consistently observed in cases of pain conditions, suggesting shared biological pathways, like inflammatory signals, immune activation and neuronal stress responses (64). These conserved signature patterns support the concept that chronic pain conditions may share common immune-neural interactions and molecular mechanisms of sensitization (64).

Additionally, sex-specific transcriptomic differences critically regulate the biology of pain, resulting in varying rates of incidence and severity between sexes. Meanwhile, transcriptomic analysis identified genes with sex-dependent expression patterns in correlation with pain states (112). Notably, T-cell leukemia/lymphoma 1A (TCL1A) showed increased expression in females, and its expression level was significantly associated with the severity of neuropathic symptoms (*r* = 0.48, *p* < 0.01), thus it can be used as a potential biomarker for neuropathic pain (111). Importantly, these findings are supported by protein-level validation in patient cohorts, strengthening the evidence that TCL1A may represent a clinically meaningful biomarker candidate (113).

Furthermore, transcriptomic analysis has been useful for identifying genes that appear to be shared across multiple chronic pain conditions. One such gene is endoplasmic reticulum aminopeptidase type 2 (ERAP2), which modulates antigen processing and immune regulation (114). The repeated identification of ERAP2 across multiple pain-associated transcriptomic datasets indicated that antigen presentation and activation of the immune system may play a significant role in the pathophysiology of chronic pain (67). These findings demonstrate that blood gene expression profiling can reveal both shared molecular mechanisms and patient-specific biological differences, like sex-dependent pathways (Table 2).

**Table 2:** Candidate biomarker gene/pathway modules (neuropathic pain–weighted).

Biomarker genes	Pathway modules	Outcomes	Refs.
TCL1A	Circulating immune cells, sex-specific prognosis	In females ↓ and significantly associated with the severity of neuropathic symptoms	(111)
ERAP2	Immune antigen processing	↑ in patient with pain	(67)
EFNB2, GABBR1, NCAM1, SCN11A	DRG-associated chronic gene enrichment	↑ in patients with chronic pain	(60, 183)
ADAR2	5-HT2CR, COPA, GluA2	↑ in rats after peripheral nerve injury	(98)
Senescene score/nociceptor senescence	Chronification mechanisms and treatment responsiveness	↑ TRPV1 + nociceptors expressed senescent	(120, 184)

### 4.2 DRG-focused human molecular context: acute vs. chronic pain signals

Recently, at single-cell resolution, single-cell sequencing has improved understanding of the composition and functional heterogeneity of human DRG neurons and has further provided detailed characterization of DRG neuronal subtypes and their gene expression profiles (115). These findings may be helpful in identifying new target genes and also developing DRG neuron cell-type specific therapies for the optimized neuropathic pain treatment (52). Studies integrating GWAS data with single-cell RNA sequencing data from DRG and enriched pain-associated variants were found mainly in the hippocampal CA1–3, prefrontal cortex, and amygdala (60). Meanwhile, mapping of genomic loci associated with chronic pain onto primate sensory neuron types can be helpful in the identification of the cellular origin of chronic pain (116). Furthermore, genes implicated in the DRG-focused analysis are EFNB2, GABBR1, NCAM1, and SCN11A, all of which have a significant role in signaling of pain, as described in Table 2 (60). The identification of these genes in transcriptomic studies suggested that molecular changes in the sensory neurons are central to the chronic pain pathogenesis (117). Notably, these neuronal signatures support the idea that clinically useful biomarkers can arise from integrating neuronal and immune molecular information.

### 4.3 Mechanism-to-biomarker translation using atlases

Despite a large number of publications reporting biomarker discovery, the FDA has reportedly approved only 1–3 new biomarkers for clinical use each year (118). This may be due to major challenges in biomarker research, which make it difficult to identify molecular signatures that are robust, generalizable, and reproducible (119). Studies addressing molecular atlas initiatives have already begun to address such challenges by integrating diverse datasets and standardizing analytical frameworks.

A prominent example of this approach is the iPain integration approach, which is used to build a comprehensive atlas of pain-associated molecular signatures by integrating data from multiple experimental models, including animal and human cohorts (120). This is crucial for the investigation of chronic pain development, particularly when originating in the first anatomical relay of pain pathways, either in the TG (iPain TG) or DRG (iPainDRG) (120). Through multi-dataset harmonization and cross-study integration, iPain provides a framework, which can be used for identification of biomarkers that consistently appear across independent datasets. This iPain integrative strategy is particularly valuable as it allows scientists to distinguish true biological signals from dataset-specific artefacts and also allow for early recognition of the disease (121).

## 5 Proposed system and method: an RNA-based “objective pain test”

The assessment of chronic pain currently relies on subjective patient-reported measures, like visual analog scales (122). This tool provides clinical insights, however, they are inherently influenced by mood, psychological state and individual perception of pain, which can introduce variability and bias in diagnosis and treatment monitoring (123). Advances in transcriptomics and molecular biology provide insights into the gene expression fingerprint of specific sensory neuronal subtypes and also provide opportunity to complement subjective assessments with objective biological indicators of mechanisms associated with pain (124). Pain biology scores are often derived from multilayer blood biomarkers that can track pain severity and are also helpful in its assessment and treatment (125).

### 5.1 System concept: “pain biology score” built from multi-layer RNA biomarkers

The primary biological input can be peripheral blood samples, such as PBMCs or whole blood, which are readily accessible in routine clinical practice (126). In addition, saliva can be considered, a non-invasive source that contains various important biological molecules and macromolecules, which can be used as biomarkers for the diagnosis of diseases, prognosis, and response towards treatments (127). In research settings, cerebrospinal fluid (CSF), skin biopsies or derived nociceptor models can provide deeper mechanistic insights (128, 129).

As a consequence, the systems generate several relevant clinical outcomes that reflect key underpinning mechanisms, which can be used for diagnosis, patient stratification, drug development, and treatment monitoring (130). For diagnostic support, this system identified molecular signatures consistent with mechanisms of pain, including inflammatory, nociceptive, central hypersensitivity, and neuropathic mechanisms (131). Likewise, for patient stratification, grouping patients according to dominant biological pathways, for instance, immune-driven phenotypes of pain, nociceptor excitability-associated mechanisms or central nervous system risk signatures (132). This system also employed strategies such as next-generation sequencing for molecular delta signatures, which enables more precise treatment monitoring by analyzing molecular profiles before and after therapeutic interventions (133, 134).

Quantitatively, the Pain Biology Score (PBS) should not rely on simple cumulative expression but must be computed using a multi-omics integration algorithm, such as a weighted logistic regression model. The overall normalized score can be expressed mathematically as:

$$PBS=\overset{n}{\underset{i=1}{\sum }}\;(\beta_{i}\;X\;E_{i})+\overset{m}{\underset{\;j=1}{\sum }}\;(\gamma_{j}\;X\;M_{j})+\overset{p}{\underset{k=1}{\sum }}\;(\delta_{k}\;X\;R_{k})$$

where *E*, *M* and *R* represent the normalized abundance of mRNA, miRNA, and RNA editing events, respectively. The coefficients (*β*, *γ*, *δ*) represent computationally derived weights optimized for cross-validated predictive accuracy against the quantitative sensory testing (QST) reference standard. This ensures the output is a standardized, continuous probability scale (e.g., 0–100) that directly correlates with risk.

### 5.2 Biomarker stack design

**Figure 2:**
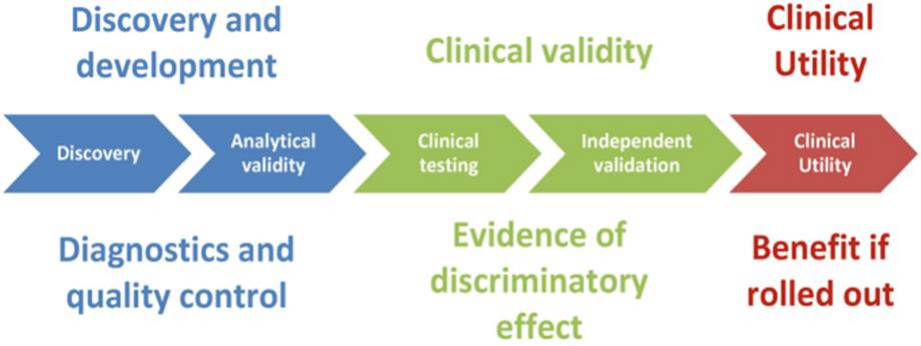
Biomarker stack design.

To capture the complexity of pain biology, the proposed system uses a tiered biomarker stack composed of complementary RNA signals (Figure 2).

#### 5.2.1 Tier 1: accessible blood transcriptomics panel

The tier 1 focuses on circulating immune gene expression, which reflects immune and systemic inflammatory processes associated with chronic pain and genes, like TCL1A and ERAP2, serve as representative biomarkers within immune-associated modules (67, 111). These biomarkers contribute to the identification of immune-related pain phenotypes and potential risk profiles. However, a primary challenge of deploying blood-based transcriptome panels is the significant temporal and phenotypic heterogeneity within patient populations over time. For instance, in conditions like diabetic neuropathic pain, patients may initially present with variable pain phenotypes but later progress to loss of sensation, potentially reflecting the progressive degeneration of sensory neurons and peripheral nociceptors. To prevent single-time-point sampling from confounding plasma RNA interpretation, future clinical study designs must integrate longitudinal serial sampling matched with longitudinal clinical phenotyping. Furthermore, cohorts should be rigidly stratified based on neurodegenerative staging and disease duration to isolate active inflammatory signaling from late-stage structural nerve loss.

#### 5.2.2 Tier 2: neuropathic pain mechanistic modules

The tier 2 focuses on the incorporation of genes linked to sensory neuron signaling and neuronal excitability, particularly those expressed in DRG neurons, and this may provide insights into the cellular and molecular basis of neuropathy (135). Based on recent research, the DRG genes, like EFNB2, GABBR1, NCAM1, and SCN11A, have been identified as key players in the chronic pain genetic architecture, particularly their role in mediating inhibitory neurotransmission, synaptic plasticity, sodium channel-mediated neuronal excitability, and cell adhesion within the central and peripheral nervous system (58, 71). Crucially, some DRG genes, such as GABBR1, NCAM1, and SCN11A, also exhibit robust expression and activity within the central nervous system (CNS). Their genetic association with chronic pain intensity may be heavily driven by central processing mechanisms. Notably, a recent multi-ancestry genome-wide association study reported that genes associated with pain intensity are largely enriched in CNS-resident tissue networks (136). Consequently, total peripheral blood RNA profiling may not completely isolate these central, CNS-driven mechanisms of pain. Given the strict practical and ethical challenges associated with obtaining spinal cerebrospinal fluid samples from patients with chronic pain, alternative non-invasive tracking strategies are required, such as enriching neural-derived blood biomarkers. So, researchers can selectively sequence CNS-derived RNA from a standard blood sample and bridge the gap between peripheral biomarkers and central pain pathology.

#### 5.2.3 Tier 3: post-transcriptional modules

The third tier involves the integration of all post-transcriptional regulatory signals, encompassing both non-coding RNAs and the broader epitranscriptomic landscape. A foundational component of this tier comprises circulating miRNA panels, which serve as stable, highly quantifiable biomarkers for diagnosis, prognosis, and treatment responsiveness (142). These regulatory RNAs govern critical alterations in neuronal excitability, synaptic transmission, and the propagation of pro-inflammatory cascades during the development of neuropathic pain (142).

Crucially, this tier also unifies RNA editing and chemical RNA modifications, as both operate post-transcriptionally to dynamically regulate pain-associated genes. A prominent mechanistic example is adenosine-to-inosine (A-to-I) RNA editing. Catalyzed by ADAR enzymes (e.g., ADAR2), this process directly generates inosine modifications on targeted transcripts. Consequently, ADAR-mediated editing events are now recognized as primary mediators of injury-induced tactile allodynia and represent promising, highly specific therapeutic targets (102, 143). Beyond inosine, this post-transcriptional tier includes a spectrum of epitranscriptomic marks, most notably N6-methyladenosine (m6A) and N1-methyladenosine (m1A), which control RNA stability and translation efficiency to optimize personalized clinical therapy (144).

Currently, directly mapping and quantifying absolute levels of these epitranscriptomic modifications (e.g., via LC-MS/MS or specialized MeRIP-seq) remains technologically challenging, expensive, and limited in routine clinical throughput. To overcome this barrier, we propose the clinical implementation of a targeted transcriptomic surrogate panel. By quantitatively profiling the mRNA expression levels of key epitranscriptomic regulators, specifically the “writers” (e.g., METTL3, METTL14), “erasers” (e.g., FTO, ALKBH5), and “editors” (e.g., ADAR1, ADAR2), researchers and clinicians can accurately infer the post-transcriptional state of the nociceptive system. This targeted regulator panel provides a highly scalable, practical methodology for studying epitranscriptomics in chronic pain, bridging the technological gap to accelerate new diagnostic and therapeutic developments.

### 5.3 Longitudinal monitoring use cases

A key advantage of RNA-based biomarkers associated with pain is their potential for longitudinal monitoring of treatment responses, given their dynamic nature and high stability in biofluids (137). Baseline measurements can be obtained before the initiation of therapies, such as opioid medication, analgesics, neuro-modulation treatments, and neuropathic pain agents.

One particularly important application is spinal cord stimulation (SCS), which is a highly effective, evidence-based neuro-modulation therapy used for the management of chronic pain (138). For instance, Liu et al. (139) reported outcomes after SCS, as 37 patients were implanted with SCS and pain was measured for 12 months. After 12 months, patients in the SCS group demonstrate improved pain relief (2.35 VAS, *p* < 0.001) and also show increased intensity of microcirculation of calves in the SCS group. Meanwhile, in animal model, after SCS, RNA sequencing was performed, and the results suggest further increases in many existing upregulated immune responses, including activation of non-neuronal cells, and transcription of cell surface receptors (140). This approach would provide an objective measure of treatment response beyond subjective pain scores. By tracking changes in molecular signatures over time, the proposed system can be helpful for clinicians to determine whether therapies are effective at targeting the biological pain drivers, ultimately supporting more patient-specific and evidence-based management strategies for pain.

## 6 Where low input RNA kits fit: enabling scale, speed, and low-input testing

The development of RNA-based biomarkers for chronic pain depends not only on the identification of relevant molecular signatures but also on the establishment of practical laboratory technologies that can easily translate discovery into routine clinical testing. A major bottleneck in this process is the current limitation in RNA analytics, particularly for advanced molecular layers such as epitranscriptomics. RNA modifications are often present in low abundance, and cellular heterogeneity makes it exceedingly difficult to differentiate cell-specific modification profiles (149). Traditional methodologies for measuring these modifications demand highly specialized equipment, complex bioinformatics workflows, and prohibitively large amounts of input RNA, severely restricting their feasibility for routine, high-throughput clinical use.

Consequently, there is an urgent, unmet clinical need for scalable, rapid, and low-input testing platforms capable of profiling minute samples (e.g., scarce neural tissues or minimal biofluid draws) without sacrificing sensitivity. To address these critical pain points, up-and-coming biotechnology companies, such as Lilac Biosciences, are pioneering specialized RNA analytical tools. By developing next-generation quantitative reagent kits and streamlined laboratory workflows (150), new workflows aim to bridge the gap between complex molecular discovery and practical application. Within the context of an objective, RNA-based pain-testing system, these low-input platforms represent an essential enabling component. By overcoming the traditional barriers of scale and sample volume, they provide the rapid epitranscriptomic profiling necessary to support a broader, multi-layer biomarker architecture for personalized pain management. However, challenges associated with standardization, regulatory approval and large-scale clinical validation remain substantial. At present, no single technology fully satisfies all requirements for routine clinical applications. The future progress of this equipment depends on the integration of complementary platforms and conducting more rigorous multicenter validation studies to establish clinically meaningful and reproducible RNA-based biomarkers for neuropathic pain.

## 7 Clinical validation roadmap

**Figure 3:**
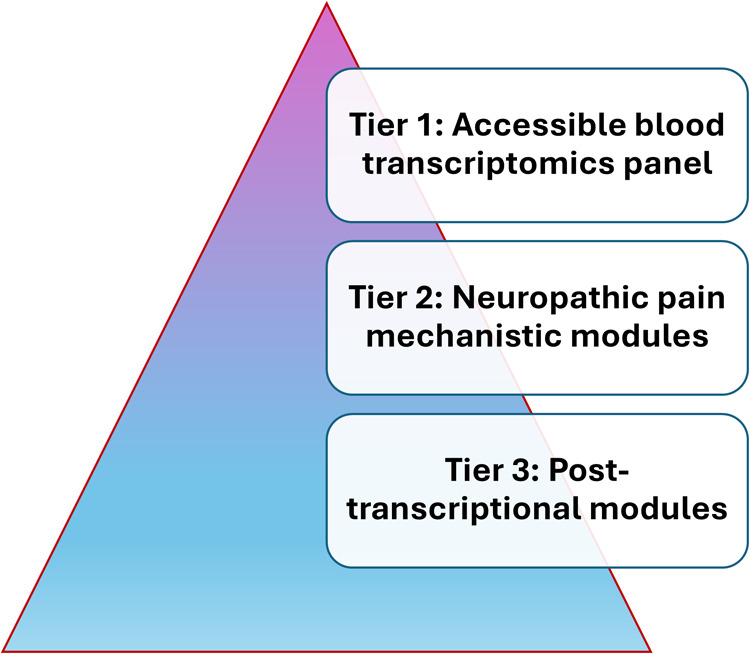
Discovery and validation steps for biomarker-guided trials (172).

The successful translation of an RNA-based analytical test into clinical practice requires a structured validation strategy that includes explainability and transparency, external validation, testing setting and context, generalizability, ease of deployment in clinical trials and settings, and cost-effectiveness (105). Because biomarkers associated with pain involve a complex molecular signature rather than single-molecule biomarkers (105). The proposed roadmap begins with analytical validation of the assay system in well-defined patient cohorts and assays regulated through laboratory-developed tests (LDTs), with optimal expansion towards FDA approval for broader utilization, as described in Figure 3 (141).

### 7.1 Analytical validity

Analytical validation aims to establish the performance characteristics of the biomarkers and reliably measures the intended molecular targets under defined laboratory conditions (142). The process can be started with conceptualization, which involves the discovery and development of biomarkers. This phase can include studies aimed at the verification of the accuracy and reliability of the methods used for detection, which are helpful in formulating a hypothesis for the context of use (COU), and in assessing the reproducibility and robustness of the assay (105). In addition, other validation parameters, such as the limit of quantification (LOQ) and limit of detection (LOD), can be used for analytical validation of the biomarkers (143). Additional validation, such as assessment and mitigation of batch effects, is critical for ensuring the reliability and reproducibility of omics data and also helpful in minimizing the impact of technical variations on biological interpretation (144). Furthermore, another method, which is useful for assessing, indirect and specific manner, whether binding of miRNA occurs to a specific predicted target mRNA (145).

Crucially, as epitranscriptomic methods transition from basic research to clinical application, they require extra attention regarding rigorous standardization and assay specificity due to their relative novelty. Assays designed to capture and quantify RNA modifications frequently rely on antibody-based enrichment (e.g., m6A-MeRIP-seq), which can be highly susceptible to off-target cross-reactivity and variable binding affinities. To successfully analytically validate these advanced biomarkers in a clinical setting, laboratories must mandate the use of precisely calibrated reference materials such as synthetic modified RNA spike-in controls and employ orthogonal validation techniques (like LC-MS/MS) to guarantee absolute molecular specificity and prevent false-positive signal amplification.

An important differentiating feature in this context is the integration of built-in standards and quality control systems, which can be incorporated directly into the workflow of the assay. Internal calibration using ratio-based transcriptome-wide reference datasets that effectively provide cross-laboratory and cross-platform ground truth. This approach enables sensitive assessment of cross-batch transcriptomic data integration at the ratio level (146). Moreover, integrating cloud-based software applications streamlines data normalization and quality monitoring. These applications can support regulatory compliance, enhance reproducibility, and trigger timely alerts for item expiration and quality control reviews (147).

When evaluating this integrated pipeline, it is crucial to recognize that different RNA modalities currently occupy distinct stages of clinical readiness. For instance, targeted mRNA expression panels utilizing multiplexed quantitative PCR (qPCR) are already deeply entrenched in clinical diagnostics. Because qPCR workflows benefit from decades of standardized protocols, absolute quantification via standard curves, and robust endogenous reference genes, they represent the most biologically feasible and immediate first step for rolling out a clinical pain biomarker test. Conversely, modalities such as circulating miRNA panels, while highly informative post-transcriptionally, are significantly more susceptible to analytical bottlenecks. miRNAs are highly sensitive to pre-analytical variations, including minor deviations in sample preparation, extraction efficiency, and biofluid hemolysis. Therefore, successfully integrating miRNA panels into this diagnostic pipeline demands highly stringent, modality-specific quality controls, such as the mandatory inclusion of synthetic exogenous spike-in RNA to strictly normalize extraction recovery rates. By stratifying these technologies and deploying the highly validated qPCR expression modules as a frontline tool, clinicians can achieve a staggered, biologically comprehensive, and clinically reliable multi-omics platform.

### 7.2 Clinical validity (neuropathic pain emphasis)

Clinical validity is the ability of the biomarker to accurately predict or identify a clinical condition. In the context of neuropathic pain, the validation is crucial for overcoming the challenges of subjective pain reporting due to its biologically distinct nature (148). Defining appropriate clinical reference standard is most crucial for this phase.

For this purpose, multi-dimensional gold standard approach, like quantitative sensory testing (QST), which is used to examine the sensory perception after application of different thermal and mechanical stimuli of controlled intensity and the function of both small (A-delta and C) and large (A-beta) nerve fibers, including corresponding central pathways (149). Another tool that can be used is patient-reported outcomes (150). Notably, the most widely used method for neuropathic pain phenotyping is the pain DETECT questionnaire, which promptly alerts clinicians that patients may need further diagnostic evaluation or therapeutic interventions, and can also predict treatment response (151). Combining these measures can provide a robust reference framework for evaluating RNA biomarker scores.

Meanwhile, clinical validation studies should include diverse cohorts of patients, representing common subtypes of neuropathic pain. Among these subtypes, they may include diabetic neuropathy, postherpetic neuralgia (PHN), trigeminal neuralgia, chemotherapy-induced peripheral neuropathy (CIPN), and CRPS neuroinflammatory condition (3, 5, 152, 153). Biomarker development should be prioritized for early diagnosis, particularly in CRPS, where prompt intervention significantly improves clinical outcomes. Including these multiple subtypes, allows researchers to determine whether the RNA-based biomarker signatures capture shared neuropathic mechanisms or disease-specific variations. Likewise, for better validation of these techniques, control or non-neuropathic chronic pain subjects should also be included, which would provide a clearer picture. This type of comparison is also important for evaluating biomarker specificity and ensuring that the molecular signatures reflect neuropathic biology rather than generalized pain or stress responses (154). Another important component of clinical validation is sex-stratified analysis, motivated by emerging evidence of sex-dependent molecular differences in disease biology (155). In case of chronic pain, sex plays a fundamental role in shaping neuroimmune mechanisms that operate across spinal, peripheral, and supraspinal levels (156). For instance, TCL1A has been identified as a potential sex-specific biomarker associated with neuropathic pain severity in females, as increased expression of TCL1A was found in females compared to males (111). Therefore, stratifying validation cohorts by sex can be helpful in determining biomarker performance across both sexes. Additionally, these preliminary findings are constrained by small sample sizes and are based on isolated discovery datasets. The clinical significance of TCL1A has not yet been validated in independent large cohorts, these findings must be interpreted with caution. Further clinical validation remains necessary before its utility as a reliable diagnostic marker can be established.

### 7.3 Clinical utility

Evaluation of the clinical utility of biomarkers requires a phased approach, as in the early phase, studies must demonstrate that the biomarkers are statistically associated with the clinical state of interest and add valuable information regarding the presence or risk of disease beyond or above the established biomarkers. In contrast, mid-phase provides information regarding the performance of the biomarkers (157). One important advantage may be the earlier identification of neuropathic pain mechanisms, allowing clinicians to differentiate the mechanisms involved in the neuropathy from centralized or inflammatory pain syndromes before symptoms fully evolve (158). Early identification of neuropathic pain via biomarkers is essential for the implementation of more targeted, precise, and mechanism-based treatments that can effectively improve patient-associated outcomes. Another advantage can be therapy selection and patient stratification based on the pathophysiological subsets of pain, and to evaluate target engagement and response of drugs (105). Biomarkers can be used to monitor the overall effect of treatment and are also helpful in the estimation of susceptibility or risk of developing the condition (159). Improved characterization of neuropathic pain using biomarkers, it allows for the identification of mechanisms that can effectively reduce ineffective trial-and-error therapy cycling (160).

### 7.4 Regulatory strategy

The initial deployment of an RNA-based biomarker system could occur through CLIA-LDT pathway, which allows clinical laboratories to develop, validate, and perform high-complexity molecular tests used for the identification of RNA profiles (161). Meanwhile, in case of broader commercialization, the platform may eventually seek FDA regulatory approval, which requires clear intended use statements, classification of risk, and demonstration of analytical and clinical performance through clinical studies (105). In addition, algorithm transparency, software validation, and governance of biomarker scoring algorithms in cases involving machine learning components are incorporated.

## 8 Applications for trials and real-world evidence

In pain research and clinical practice, integrating RNA-based biomarkers can offer significant opportunities in improving clinical trial design, real-world treatment strategies, and the evaluation of therapeutic interventions. Chronic pain studies often face limitations, such as large placebo responses, heterogeneous populations, and difficulty in measuring objective biological changes (79). RNA-based biomarkers or molecular signatures can be helpful in addressing these limitations by providing measurable indicators of underlying biology of pain and treatment response.

### 8.1 Drug trials

One of the main applications of RNA-based biomarkers is in the development of drugs and clinical trials by improving patient stratification, target validation and assessment of efficiency (105). RNA-based biomarkers, such as lncRNA dysregulation, significantly impact the perception of pain by affecting pain-associated mechanisms, including neuro-inflammation and neuronal excitability (162). Meanwhile, traditional clinical trials often struggle to demonstrate treatment effects because included patients may have heterogeneous biological pain mechanisms (163). To overcome this limitation, RNA-based biomarkers can offer a solution, as in this approach, patients were included based on molecular signatures indicating biomarker-positive neuropathic biology (79). By including patients whose molecular profiles match the biological target of the therapy, researchers can increase the likelihood for the detection of meaningful treatment effects and improve statistical power. In addition, RNA-based biomarkers are highly dynamic, making them ideal pharmacodynamic readouts for monitoring the response to treatment, progression of disease and early therapeutic efficacy (164). In this context, numerous RNA-based biomarkers may be particularly useful.

Among RNA-based biomarkers, one category involves miRNA changes, which reflect regulatory shifts in the expression of gene networks associated with neuronal signaling, inflammation and stress responses (165). Certain circulating miRNAs have demonstrated changes in their level during chronic pain and normalize after effective treatment (137). Another important and promising pharmacodynamic marker involves RNA editing signatures, particularly those associated with ADAR2-mediated editing events in neuropathic pain (98). Monitoring ADAR2 could therefore provide insights into whether it has therapeutic potential and whether it can be successfully targeted for the treatment of neuropathic pain (98).

Furthermore, expression module shifts are another increasingly recognized as critical pharmacodynamic signals that reflect the molecular response of tissue to treatment, particularly in the context of pain (166). Transcriptomic modules, which involve DRG-associated genes may change in response to treatment therapies (166). During treatment, tracking these expression module changes can be helpful for researchers to determine whether a drug is producing the expected molecular effects.

### 8.2 Neuro-modulation trials and device efficacy

Another crucial application of RNA biomarker systems lies in the assessment of neuro-modulation therapies, particularly SCS used for chronic neuropathic pain (167). These biomarkers could provide a supportive biological signal of efficacy, which can be helpful in distinguishing true physiological treatment effects from placebo-associated symptom improvements (168). For instance, measuring changes in the biomarker profile pre and post SCS intervention could reveal that the intervention alters the biological pathways associated with neuropathic pain (169). Another most important piece of evidence was provided by Vallejo, Kelley (170), who demonstrated that SCS, a neuro-modulation therapy, can effectively modulate multiple, complex biological processes beyond pain pathways, returning their expression to levels observed in non-injured and naïve animals (170). Therefore, RNA-based biomarkers can be used to test specific mechanistic hypotheses regarding how neuro-modulation therapies, such as SCS, exert their therapeutic effects. Meanwhile, reduction in immune-related transcriptomic signatures could indicate dampening of inflammatory pathways and can alter neuronal activity in ASD temporal cortex (171). By linking molecular signatures with clinical outcomes, systems like RNA-based biomarkers can provide valuable insights into the patient-specific response pattern and mechanisms of action for neuro-modulation therapies. Moreover, beyond clinical trials, blood biomarker systems can also provide valuable information and support precision pain medicine approaches in a routine clinical practice (125).

## 9 Limitations, pitfalls, and how to address them

RNA-based biomarkers have promising potential for objective pain assessment, however, several limitations need to be acknowledged. One important limitation in the translation of RNA biomarkers into clinical practice is balancing biological complexity with practical applicability in the development of drugs. Although neuropathic pain is increasingly recognized as a condition which is characterized by multidimensional molecular dysregulation rather than a single pathogenic pathway, the pharma industry often focuses on target-specific biomarkers that can be efficiently incorporated into clinical trials. While integrating pain biology scores and multiple RNA-based signatures may provide a more comprehensive representation of disease mechanisms, patient heterogeneity, and treatment response. However, the development of such score systems requires large-scale validation across diverse geographical populations, standardized analytical pipelines, and clinical settings. The requirements may exceed the capabilities of the pharma industry. Consequently, the establishment of validated pain biology scores may be better suited to international collaborative consortia that can bring the pharma industry, academic researchers, patient registries, and regulatory stakeholders. Such integration can successfully facilitate external validation, data harmonization, and standardization of biomarkers, resulting in a robust platform that pharma companies can subsequently employ for patient stratification, assessment of target engagements, and monitoring of the treatment. Another important limitation is the accessibility of tissues, as the biological mechanisms underlying chronic pain often originate in DRG, CNS and peripheral nerves. However, in routine clinical practice, these tissues are not readily accessible. As a result, most biomarkers rely on peripheral blood samples, including PBMCs. Despite the fact that blood-based transcriptomics provides a practical advantage in terms of scalability and accessibility, it may not fully capture the molecular events occurring directly within neuronal tissues. For these reasons, signals from blood neuronal pain mechanisms need to be interpreted with caution. Meanwhile, to overcome accessibility issues, advanced analytical approaches, such as cell-type deconvolution and integrative phenotype mapping, are necessary. Another limitation involves the presence of confounding biological and environmental factors [infection, autoimmune disease, medicines, circadian rhythms, smoking, body mass index (BMI)] that can influence RNA expression profile. These factors may alter epitranscriptomic and transcriptomic signatures independently of pain biology. Mitigating these confounding factors requires careful study design and data normalization strategies. Validation studies performed clinically should include comprehensive patient meta-data to allow adjustment for potential confounding factors during statistical analysis. In addition, incorporating multivariate models and reference control cohorts may also be helpful in isolating pain-specific molecular signals from broader physiological influences. Further limitations arise from risks associated with overfitting and dataset shift, particularly when biomarker models are developed using complex multi-marker algorithms. Therefore, rigorous multi-site validation and independent cohort replication are required to address these concerns. Lastly, ethical considerations must be carefully addressed when introducing objective molecular tests for pain assessment. It is essential to position RNA-based biomarkers as complementary tools rather than replacements for clinical assessment.

While the multi-tiered RNA biomarker architecture offers profound insights into neuropathic pain chronification, transitioning these modalities from discovery to clinical utility requires careful mitigation of distinct analytical bottlenecks. Each RNA class presents unique technical and biological challenges that must be addressed to ensure diagnostic reproducibility.

### 9.1 Circulating RNAs and miRNAs

Despite their clinical promise, circulating RNAs, particularly miRNAs, are highly susceptible to both pre-analytical and analytical variables. They are acutely sensitive to inconsistencies in sample collection, handling protocols (e.g., biofluid hemolysis), and variable *ex-vivo* stability. In longitudinal studies, these factors often introduce significant batch-to-batch variability. Furthermore, their inherently low abundance in plasma or serum makes accurate reverse transcription and PCR (RT-PCR) amplification technically demanding. This low target density severely complicates the identification of stable endogenous reference controls, making robust cross-cohort normalization a persistent challenge.

### 9.2 Long non-coding RNAs (lncRNAs)

Similarly, the clinical quantification of lncRNAs faces significant translational hurdles. Unlike highly conserved protein-coding transcripts, lncRNAs frequently exhibit profound person-to-person biological variance, complicating the establishment of universal baseline thresholds for healthy vs. neuropathic states. Additionally, the lack of universally accepted, stable “housekeeping” lncRNAs exacerbates normalization challenges, particularly when comparing heterogeneous neural tissues or distinct patient demographics.

### 9.3 Epitranscriptomics and RNA modifications

Conversely, the quantification of RNA modifications (such as m6A or inosine) using gold-standard techniques like liquid chromatography-tandem mass spectrometry (LC-MS/MS) offers a distinct advantage: these modifications can be measured as absolute stoichiometric ratios (e.g., m6A/A) that are conserved per cell, inherently solving many normalization issues. However, the modality is severely bottlenecked by current technical limitations. Traditional epitranscriptomic profiling demands prohibitively high RNA input requirements, extensive analytical lead times, and high costs that currently preclude high-throughput, routine clinical use.

### 9.4 Closing the analytical gap

To realize a truly integrated, multi-omics pain diagnostic platform, these methodological barriers must be overcome. Fortunately, emerging biotechnology companies, such as Lilac Biosciences, are actively engineering solutions to close these analytical gaps. By developing specialized, rapid, low-input quantitative workflows and standardized reagent kits, these platforms aim to bypass traditional assay limitations ensuring that complex measurements like RNA modifications can feasibly transition from basic research into robust, routine clinical practice.

## 10 Future directions

In future directions, one of the most promising directions is the continued development of atlas-driven biomarker expansion (iPain-style integrative atlases for selecting conserved markers). These large integrative datasets combine single-cell sequencing, transcriptomics, and genomic data across tissues, and can be helpful in the identification of conserved molecular signals associated with chronic pain.

Another important future direction is strengthening the connection between molecular biomarkers and the underlying mechanisms involved in pain. Currently used transcriptomic panels can identify associations between clinical pain phenotypes and RNA signatures, however, a deeper understanding can further improve the predictive and therapeutic value of these biomarkers. One emerging concept is nociceptor senescence, a state in which sensory neurons exhibit stress-associated molecular changes, leading to the persistence of chronic pain and senescent nociceptors that may display altered inflammatory signals.

Furthermore, biomarker systems are likely to evolve through multi-omics integration by combining information from multiple molecular layers used for the generation of more comprehensive biological profiles. This integration may significantly improve the accuracy and interpretability of molecular pain phenotyping.

Additionally, cerebrospinal fluid (CSF)-derived biomarkers and exosomal RNA-based biomarkers, highlight its potential in oncological research. In pain research, it can also play important role, future studies need to address the potential of these CSF and exosomal biomarkers.

There is also a need to address reproducibility challenges via standardized methodologies, multicenter collaborations, and independent validation studies. Future studies should also investigate the subtype-specific RNA signatures across different neuropathic pain conditions, such as postherpetic neuralgia, diabetic neuropathy, or other conditions.

Another important future direction is the ethical consideration, which can accompany the clinical implementation of RNA-based diagnosis of neuropathic pain.

Lastly, the introduction of more standardized endpoints could improve the interpretability and consistency of clinical trials.

## 11 Conclusion

RNA-based biomarkers represent a promising step towards the development of more objective and biologically informed tools for the diagnosis and treatment of neuropathic pain. Emerging evidence from transcriptomic studies, including mRNA, miRNA, lncRNA, epitranscriptomic modifications, including RNA editing suggests that molecular signatures can capture mechanisms underlying pain biology. Integration of these multi-layer RNA signals into a structure-based biomarker framework may be useful for improved patient stratification, supporting mechanism-based selection of treatment and providing objective measures for monitoring therapeutic response. However, its validation remains. Therefore, future studies should focus on validation, including the inclusion of multi-site validation, continued advances in molecular atlases, and multi-omics integration, which may accelerate the translation of RNA-based biomarkers into clinical practice.
